# Impact of the adequacy of antibiotic therapy on the outcome of ventilator-associated pneumonia

**DOI:** 10.1186/cc9640

**Published:** 2011-03-11

**Authors:** J Goncalves-Pereira, T Sequeira, B Moya, T Cardoso, N Catorze

**Affiliations:** 1 Hospital Sao Francisco Xavier, Lisboa, Portugal; 2 Hospital Sao Jose, Lisboa, Portugal; 3 Hospital Sao Bernardo, Setubal, Portugal; 4 Hospital Santo António, Porto, Portugal; 5 Centro Hospitalar Médio Tejo, Abrantes, Portugal

## Introduction

The aim was to assess the impact of empiric antibiotic adequacy on ICU outcome of patients with ventilator-associated pneumonia (VAP), the reasons for inadequacy and risk factors for potential multidrug-resistant organisms.

## Methods

During a 24-month period a multiple-centre observational study was conducted in five ICUs. Adult patients with documented VAP were segregated for analysis. Empiric antibiotic therapy was classified as adequate or inadequate according to *in vitro *efficacy against all isolated bacteria. The day of ICU discharge or death was recorded. Comparison between survivors and nonsurvivors was performed. Infection with potential multidrug-resistant organisms (methicillin-resistant *Staphylococcus aureus*, *Pseudomonas aeruginosa*, *Acinetobacter baumanii *or *Stenotrophomonas maltophilia*) was evaluated for therapeutic inadequacy, ICU length of stay before diagnosis and previous use of antibiotics.

## Results

One hundred and twenty-three patients with VAP (age 62.7 ± 16.9 years, 65.9% men, and SAPS II 49.5 ± 15.5) were identified. Empiric antibiotic therapy was adequate in 65.9%. These patients' ICU mortality was significantly lower in comparison with those with inadequate therapy (28.4% vs. 45.2%, *P *= 0.049). Patients infected with a potential multidrug-resistant organism were more likely to receive inadequate antibiotic therapy (80.1%, *P *= 0.001), and to have had longer previous ICU stay (11.5 days vs. 7.2 days, *P *= 0.005), but there was no difference in the previous use of antibiotics (65.2% vs. 50%, *P *= 0.102).

## Conclusions

An empiric adequate antibiotic therapy was associated with a lower mortality rate in VAP. Multidrug-resistant organisms were significantly associated with therapeutic inadequacy and longer ICU length of stay.

